# Efficacy and Safety of Ocedurenone: Subgroup Analysis of the BLOCK-CKD Study

**DOI:** 10.1093/ajh/hpad066

**Published:** 2023-07-20

**Authors:** George L Bakris, Y Fred Yang, James M McCabe, Jin Rong Liu, Xiaojuan J Tan, Vincent J Benn, Bertram Pitt

**Affiliations:** Department of Medicine, American Heart Association Comprehensive Hypertension Center, The University of Chicago Medicine, Chicago, Illinois, USA; Chief Development Officer, KBP BioSciences USA Inc., Princeton, New Jersey, USA; Chief Development Officer, KBP BioSciences USA Inc., Princeton, New Jersey, USA; Chief Development Officer, KBP BioSciences USA Inc., Princeton, New Jersey, USA; Chief Development Officer, KBP BioSciences USA Inc., Princeton, New Jersey, USA; Chief Development Officer, KBP BioSciences USA Inc., Princeton, New Jersey, USA; Department of Medicine, University of Michigan School of Medicine, Ann Arbor, Michigan, USA

**Keywords:** blood pressure, chronic kidney disease, hypertension, mineralocorticoid receptor antagonist, potassium, systolic blood pressure, uncontrolled hypertension

## Abstract

**BACKGROUND:**

Ocedurenone (KBP-5074), a nonsteroidal mineralocorticoid receptor antagonist, is documented to lower blood pressure in patients with stage 3b/4 chronic kidney disease (CKD) with uncontrolled or resistant hypertension (BLOCK-CKD study). However, the efficacy and safety of Ocedurenone in subgroups such as Hispanic patients or those with stage 4 CKD, diabetes, or very high albuminuria have not been reported.

**METHODS:**

A total of 162 patients were enrolled in the BLOCK-CKD study. The primary endpoint of these analyses was change in systolic blood pressure (SBP) from baseline to day 84. Prespecified subgroup analysis of SBP focused on demographic (e.g., ethnicity, age) and medical (e.g., CKD stage, diabetes, albuminuria, baseline estimated glomerular filtration rate [eGFR]). The safety analysis focused on changes in serum potassium levels from baseline.

**RESULTS:**

SBP reductions were consistent across subgroups compared with the overall study cohort. Placebo-adjusted SBP reductions were observed in Hispanic patients (−8.1 and −9.9 mm Hg for 0.25 and 0.5 mg, respectively, total *n* = 35) and patients with CKD stage 4 (−9.3 and −10.4 mm Hg for 0.25 and 0.5 mg, respectively, total *n* = 64), diabetes (−6.9 and −11.6 mm Hg for 0.25 and 0.5 mg, respectively, total *n* = 51), and very high albuminuria (−13.1 and −12.3 mm Hg for 0.25 and 0.5 mg, respectively, total *n* = 85). Changes in serum potassium were similar across all patient subgroups regardless of baseline eGFR, diabetes status, or degree of proteinuria. No cases of hyperkalemia required intervention or resulted in study discontinuation.

**CONCLUSIONS:**

Ocedurenone consistently reduced in SBP in all patient subgroups. Moreover, while small elevations in serum potassium occurred, they were not associated with Ocedurenone or study discontinuation.

## Graphical Abstract

**Figure:**
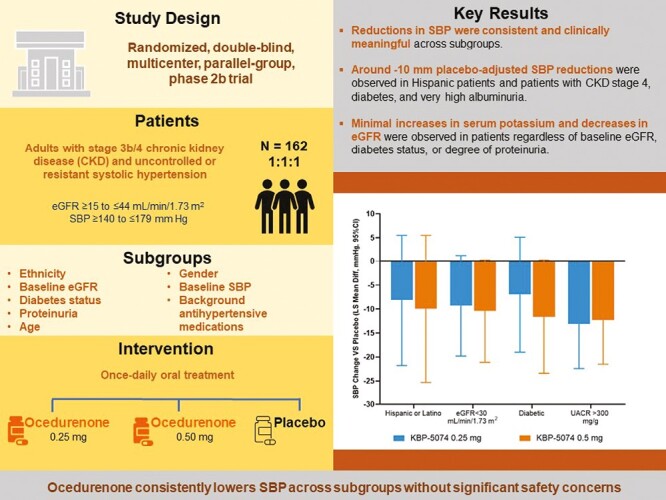

Patients with chronic kidney disease (CKD) are at increased risk of uncontrolled/resistant hypertension, which is defined as hypertension that responds poorly to treatment and requires multiple medications to reach normal blood pressure ranges.^1^ Uncontrolled/resistant hypertension is reported in about 23% of patients with CKD,^2^ and is particularly prevalent in patients with stage 4 CKD (estimated glomerular filtration rate [eGFR] 15–29 ml/min/1.73 m^2^).^3,4^

Ocedurenone (KBP-5074), a selective nonsteroidal mineralocorticoid receptor antagonist (MRA), is under development for the treatment of uncontrolled/resistant hypertension in patients with CKD.^5^ In a phase 2b, multicenter, randomized, double-blind, placebo-controlled, parallel-group clinical trial (BLOCK-CKD study, NCT03574363), Ocedurenone was associated with a clear, dose-dependent reduction of trough-cuff seated systolic blood pressure (SBP) from baseline to study day 84 compared with placebo in patients with advanced CKD and uncontrolled/resistant hypertension.^6^ Although there was an increased incidence of mild hyperkalemia, there were no instances of severe hyperkalemia (serum potassium ≥6.0 mmol/l) or acute kidney injury during the study.^6^

The objectives of this prespecified subgroup analysis of the BLOCK-CKD study were (i) to evaluate the consistent effect of Ocedurenone on SBP in various subgroups, including Hispanic patients and patients with stage 4 CKD, diabetes, or very high albuminuria; and (ii) to evaluate any changes in serum potassium levels associated with Ocedurenone use in these subgroups.

## METHODS

### Study design

The BLOCK-CKD study was a phase 2b, international, multicenter, randomized, double-blind, placebo-controlled, parallel-group study. It evaluated the safety, efficacy, and pharmacokinetics of the nonsteroidal MRA Ocedurenone for uncontrolled/resistant hypertension in patients with stage 3b/4 CKD already receiving background antihypertensive medications. The rationale and design of this study as well as details of the inclusion and exclusion criteria have been published.^7^ Briefly, female and male patients aged 18–85 years with body mass index of 19–45 kg/m^2^, stage 3b/4 CKD (defined by eGFR ≥15 to ≤44 ml/min/1.73 m^2^), and uncontrolled stage 1 or 2 systolic hypertension (defined as resting trough-cuff seated SBP ≥140 to ≤179 mm Hg^8^ at screening and the end of the placebo run-in period) were enrolled. Blood pressure measurement was performed according to American Heart Association guidelines and has been described previously.^3,7^ Patients were concurrently receiving ≥2 antihypertensive medications (i.e., diuretics, renin–angiotensin system blockers, or calcium channel blockers) at stable doses for 30 days before randomization, per guidelines.^3^ The basis for this subgroup analysis was to assess if any unique or substantive findings were observed among the older, Hispanic, and Asian patients. The sample of African Americans was too small to make a comment.

### Statistical analysis

The prespecified subgroup efficacy analysis was performed using 2-way ANCOVA models with change in trough-cuff seated SBP from baseline to day 84 as the outcome variable, treatment group as a factor, and baseline SBP value as a covariate. Depending on the subgroup analyzed, age group (≤65 years, >65 years), baseline eGFR level (≥30 ml/min/1.73 m^2^, <30 ml/min/1.73 m^2^), background antihypertensive medication (≥3 background antihypertensive medications, ≤2 background antihypertensive medications), gender (female, male), proteinuria group (very high proteinuria: urinary albumin creatinine ratio [UACR] >300 mg/g, microalbuminuria: UACR 30–300 mg/g, normoalbuminuria: UACR <30 mg/g), ethnicity (Hispanic or Latino, not Hispanic or Latino), or diabetes status (diabetic or nondiabetic based on investigator determination of cause of CKD), as well as treatment group-by-subgroup (age, baseline eGFR, background antihypertensive medication, proteinuria, ethnicity, or diabetes status) interaction were included as factors to determine whether there were differences among treatment groups within each subgroup.

For the safety analyses, descriptive statistics for changes in serum potassium across the study period from baseline to study day 84 by treatment group and by ethnic group, baseline eGFR, diabetes status, and baseline UACR are presented.

## RESULTS

A total of 162 patients were randomized: 51 to the Ocedurenone 0.25 mg group, 54 to the Ocedurenone 0.5 mg group, and 57 to the placebo group. One hundred thirty-eight of 162 patients (85.2%) completed the study, including 47 in the Ocedurenone 0.25 mg group, 44 in the Ocedurenone 0.5 mg group, and 47 in the placebo group. Eight patients (1 taking Ocedurenone 0.25 mg, 4 taking Ocedurenone 0.5 mg, and 3 taking placebo) withdrew due to adverse events, and 2 deaths occurred (both in the placebo group).

Demographics were similar across ethnic groups (Table 1). Patients had a mean age of 65.4 years and were 45.1% female, 92.0% White, and 21.6% Hispanic. A total of 64 patients (39.5%) had stage 4 CKD, 51 patients (31.5%) had diabetes, and 125 patients (77.2%) had albuminuria defined by UACR ≥30 mg/g with 85 (52.5%) patients having very high albuminuria. Randomized patients had a mean baseline SBP of 155.3 mm Hg and diastolic blood pressure of 87.7 mm Hg. Among patients with proteinuria, median baseline UACR was 1,003.7 mg/g in the Ocedurenone 0.25 mg group, 558.9 mg/g in the Ocedurenone 0.5 mg group, and 611.9 mg/g in the placebo group. Mean baseline eGFR was 31.9 ml/min/1.73 m^2^, and 144 patients (88.9%) were taking ≥3 antihypertensive medications. Baseline serum potassium was similar across treatment groups with mean 4.38 mmol/l.

**Table 1.** Baseline characteristics

	Placebo (*n* = 57)	Ocedurenone 0.25 mg (*n* = 51)	Ocedurenone 0.5 mg (*n* = 54)	Total (*n* = 162)
Age (y), mean (SD)	65.9 (10.64)	65.4 (10.76)	64.8 (13.04)	65.4 (11.47)
18 to ≤45, *n* (%)	2 (3.5)	2 (3.9)	6 (11.1)	10 (6.2)
>45 to ≤65, *n* (%)	25 (43.9)	21 (41.2)	18 (33.3)	64 (39.5)
>65 to ≤75, *n* (%)	19 (33.3)	17 (33.3)	17 (31.5)	53 (32.7)
>75, *n* (%)	11 (19.3)	11 (21.6)	13 (24.1)	35 (21.6)
Gender, *n* (%)				
Female	23 (40.4)	27 (52.9)	23 (42.6)	73 (45.1)
Male	34 (59.6)	24 (47.1)	31 (57.4)	89 (54.9)
Ethnicity, *n* (%)				
Hispanic or Latino	15 (26.3)	12 (23.5)	8 (14.8)	35 (21.6)
Not Hispanic or Latino	42 (73.7)	39 (76.5)	46 (85.2)	127 (78.4)
Race, *n* (%)				
Black or African American	2 (3.5)	5 (9.8)	5 (9.3)	12 (7.4)
White	54 (94.7)	46 (90.2)	49 (90.7)	149 (92.0)
Other	1 (1.8)	0	0	1 (0.6)
BMI, kg/m^2^, mean (SD)	30.51 (5.51)	30.81 (5.17)	31.08 (5.81)	30.79 (5.48)
eGFR, ml/min/1.73 m^2^, mean (SD)	31.6 (9.60)	31.9 (10.47)	32.2 (9.85)	31.9 (9.90)
≥30 ml/min/1.73 m^2^, *n* (%)	32 (56.1)	31 (60.8)	35 (64.8)	98 (60.5)
<30 ml/min/1.73 m^2^, *n* (%)	25 (43.9)	20 (39.2)	19 (35.2)	64 (39.5)
SBP, mm Hg, mean (SD)	155.8 (10.84)	154.3 (15.07)	155.7 (14.75)	155.3 (13.55)
DBP, mm Hg, mean (SD)	85.9 (11.46)	89.0 (12.17)	88.4 (13.06)	87.7 (12.23)
UACR, mg/g, mean (SD)	1,073.93 (1,599.59)	855.25 (1,066.65)	1,031.94 (1,361.29)	990.06 (1,361.37)
Macroalbuminuria, *n* (%)	29 (50.9)	27 (52.9)	29 (53.7)	85 (52.5)
Microalbuminuria, *n* (%)	15 (26.3)	9 (17.6)	16 (29.6)	40 (24.7)
Normoalbuminuria, *n* (%)	11 (19.3)	15 (29.4)	9 (16.7)	35 (21.6)
Potassium, central, mmol/l, mean (SD)	4.43 (0.36)	4.33 (0.38)	4.37 (0.45)	4.38 (0.40)
Potassium, local, mmol/l, mean (SD)	4.36 (0.33)	4.24 (0.39)	4.22 (0.39)	4.28 (0.37)
Serum creatinine, µmol/l, mean (SD)	186.13 (62.39)	181.99 (65.08)	185.27 (66.31)	184.54 (64.19)
Diabetes status, *n* (%)				
Diabetic	20 (35.1)	15 (29.4)	16 (29.6)	51 (31.5)
Nondiabetic	37 (64.9)	36 (70.6)	38 (70.4)	111 (68.5)
Background antihypertensive medications, *n* (%)				
≥3	51 (89.5)	44 (86.3)	49 (90.7)	144 (88.9)
≤2	6 (10.5)	7 (13.7)	5 (9.3)	18 (11.1)

Abbreviations: BMI, body mass index; DBP, diastolic blood pressure; eGFR, estimated glomerular filtration rate; *n*, number of patients; SBP, systolic blood pressure; UACR, urine albumin-to-creatinine ratio.

**Figure 1.**
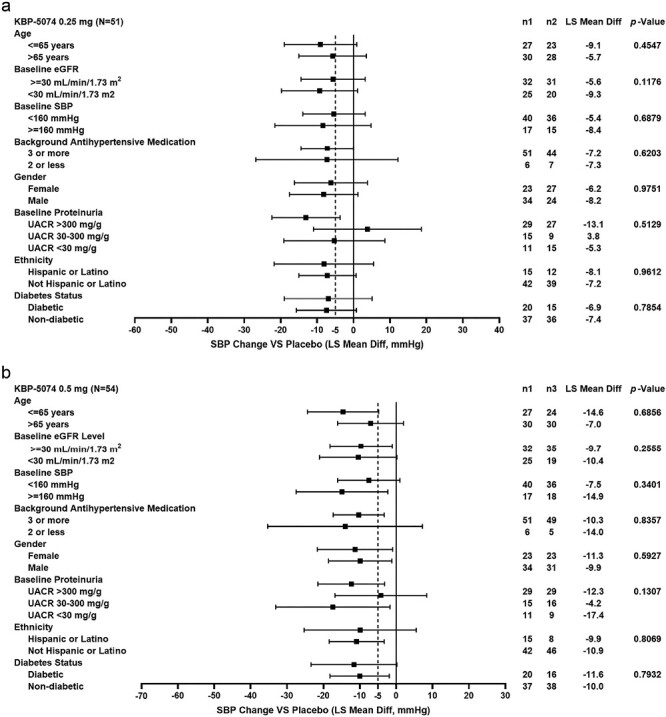
Subgroup analyses for placebo-corrected mean change from baseline to day 84 in SBP for Ocedurenone treatment groups (**a**: 0.25 mg, **b**: 0.5 mg) in intention-to-treat population. Abbreviations: eGFR, estimated glomerular filtration rate; LS Mean Diff, least squares mean difference; *n*1, number of patients on placebo; *n*2, number of patients on Ocedurenone 0.25 mg once daily; *n*3, number of patients on Ocedurenone 0.5 mg once daily; SBP, systolic blood pressure; UACR, urinary albumin-to-creatinine ratio; VS, versus.

Subgroup analyses for placebo-corrected change in SBP from baseline to day 84 are shown in Figure 1. As shown, consistent and clinically meaningful (>5 mm Hg) reductions in SBP from baseline were observed across various subgroups.

**Figure 2.**
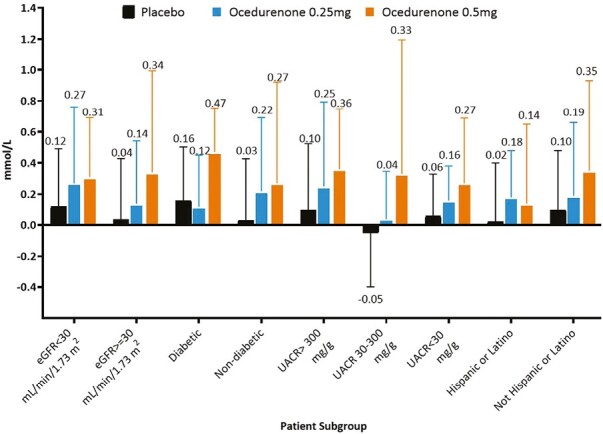
Change in potassium in mmol/l from baseline to study day 84. Data shown as mean (SD). Abbreviations: eGFR, estimated glomerular filtration rate; UACR, urinary albumin-to-creatinine ratio.

Specifically, placebo-adjusted SBP reductions were observed in Hispanic patients (−8.1 and −9.9 mm Hg for 0.25 and 0.5 mg, respectively) and patients with CKD stage 4 (−9.3 and −10.4 mm Hg for 0.25 and 0.5 mg, respectively), diabetes (−6.9 and −11.6 mm Hg for 0.25 and 0.5 mg, respectively), and very high albuminuria (−13.1 and −12.3 mm Hg for 0.25 and 0.5 mg, respectively). Changes in serum potassium across different ethnic groups, CKD stages, diabetic status, and albuminuria severity were minor, consistent, and dose-dependent across patient subgroups (Figure 2). Of note, changes in eGFR in subgroups were similar to the overall population and were not associated with changes in serum potassium levels (data not shown).

## DISCUSSION

The use of steroidal MRAs is low in patients with uncontrolled/resistant hypertension who have CKD and/or diabetes,^9,10^ and they are rarely prescribed to patients with CKD and hypertension.^11^ The nonsteroidal MRAs have a lower risk for hyperkalemia^12^ and Ocedurenone is noted to have significant blood pressure-lowering capabilities. While our observations require further confirmation, they suggest that Ocedurenone might be a more efficacious and safer option for the management of uncontrolled/resistant hypertension in patients with advanced CKD than current recommended care.^3^ Current clinical practice unfortunately demonstrates that after hyperkalemia occurs, agents that inhibit the renin–angiotensin–aldosterone system are promptly discontinued without any attempt to educate patients on low-potassium diets or use of newer, better-tolerated potassium binders, which leads to a loss of cardiorenal protection.^13^ Thus, it is critical to demonstrate efficacy and safety of a new class of drugs in patient subgroups with advanced CKD and uncontrolled or resistant hypertension due to the limited data in this population, especially patients with advanced CKD stage 4.

This prespecified subgroup analysis of the phase 2b BLOCK-CKD study demonstrates that Ocedurenone reduces SBP without major safety concerns across a variety of subgroups of patients with stage 3b/4 CKD. Specifically, this analysis provides new important information on patients with advanced CKD, including differences in ethnicity, diabetic status, stage of CKD, and severity of albuminuria. The results from this *post hoc* analysis demonstrate that Ocedurenone can lower blood pressure effectively with a minimal risk of hyperkalemia and other adverse effects across important subgroups with uncontrolled/resistant hypertension.

In the PATHWAY-2 study, spironolactone reduced SBP by 9 mm Hg compared with placebo and was superior to doxazosin and bisoprolol in patients with uncontrolled/resistant hypertension and stage 1–3a CKD, with a serum potassium level ≥6.0 mmol/l in 6 patients (2%) among people with good estimated GFRs (mean eGFR was 91.6 ml/min/1.73 m^2^).^14^ In contrast, the BLOCK-CKD study found a larger magnitude of SBP reduction with Ocedurenone in patients with uncontrolled/resistant hypertension and moderate-to-severe CKD (−10.6 mm Hg placebo-subtracted with the 0.5 mg dose) with no instances of serum potassium ≥6.0 mmol/l in patients with mean eGFR of 31.9 ml/min/1.73 m^2^.^6^ Consistent with these results, the present subgroup analysis found clinically meaningful SBP reductions in high-risk patients, which has not been previously reported.

A recent subgroup analysis of the AMBER study, where spironolactone was again used, found SBP reductions of 6 mm Hg (non-placebo-subtracted) in patients with an eGFR 25 to <30 ml/min/1.73 m^2^ (stage 4 CKD) and 12 to 13 mm Hg (non-placebo-subtracted) in patients with an eGFR 30 to 45 ml/min/1.73 m^2^ (stage 3b CKD).^15^ The AMBER study excluded patients with an eGFR <25 ml/min/1.73 m^2^, while the BLOCK-CKD study included patients with eGFR as low as 15 ml/min/1.73 m^2^. Although a direct comparison between spironolactone and Ocedurenone is not currently available, these changes in SBP are less than those seen in the BLOCK-CKD study.

Given the promising results of the BLOCK-CKD study, additional research such as the ongoing phase 3 CLARION-CKD study (NCT04968184) will help to further elucidate the efficacy, safety, and tolerability of Ocedurenone in adults with stage 3b/4 CKD and uncontrolled or resistant hypertension.

The primary limitation of this subgroup analysis of BLOCK-CKD study is the small number of patients included. This small sample size limits the generalizability of these findings. An additional limitation of this study is the relatively short follow-up period; studies with a longer duration of treatment should provide additional information about the longer-term efficacy and safety of Ocedurenone in patients with advanced CKD and uncontrolled or resistant hypertension.

We conclude that while standard care for CKD mandates use of angiotensin-converting enzyme inhibitors or angiotensin receptor blockers, the addition of a safe nonsteroidal MRA can help mitigate CKD progression and treat hypertension caused by aldosterone activation of mineralocorticoid receptors. In this subgroup analysis of the BLOCK-CKD trial, treatment with the nonsteroidal MRA Ocedurenone demonstrated consistent and clinically meaningful reductions in trough-cuff seated SBP in all subgroups studied.
